# Diabetes mellitus carries a risk of esophageal cancer: A meta-analysis

**DOI:** 10.1097/MD.0000000000007944

**Published:** 2017-09-01

**Authors:** Bo Xu, Xiufang Zhou, Xiaohong Li, Chaoyang Liu, Caizhe Yang

**Affiliations:** Department of Endocrinology, Air Force General Hospital of PLA, Beijing, China.

**Keywords:** diabetes mellitus, esophageal adenocarcinoma (EAC), esophageal cancer, meta-analysis

## Abstract

**Background:**

Many studies have showed that diabetes mellitus (DM) might be a risk factor for certain types of cancers. However, there are still inconsistent results on the effects of DM on the risk of esophageal cancer (EC). The objective of this study is to investigate the association and to quantify the correlation between DM and EC by a meta-analysis.

**Methods:**

The initial search identified 339 articles. Those publications that did not report the exact number of EC cases were removed. Finally, 13 meaningful studies were extracted from the databases of PubMed, MEDLINE, and Web of Science. All pooled analyses of risk ratios (RRs) and 95% confidence intervals (CIs) were assessed by a random-effect or fixed-effect model. Subgroup analysis was implemented on the basis of the sex or ethnicity. *I*^2^ value was used to assess heterogeneity, and funnel plot analysis was for publication bias.

**Results:**

The result showed that there was a positive correlation between type 2 diabetes mellitus (T2DM) and EC risk (RR = 1.28, 95% CI: 1.12–1.47, *P* < .001). Subgroup analysis based on gender showed that male was an important risk factor for EC (RR = 1.53, 95% CI: 1.44–1.62, *P* < .001), but female was not (RR = 1.23, 95% CI: 0.41–3.69, *P* = .71). In addition, subgroup analysis based on ethnicity showed that DM was significantly correlated to EC in North America subjects (RR = 1.39, 95% CI: 1.31–1.47, *P* < .001), and in Europe subjects (RR = 1.37, 95% CI: 1.02–1.83, *P* = .04), whereas no correlation was found in Asian subjects (RR = 0.98, 95% CI: 0.50–1.95, *P* = .96). Furthermore, DM had a correlation to an increased risk of esophageal adenocarcinoma (EAC) (RR = 1.43, 95% CI: 1.35–1.51, *P* < .001).

**Conclusion:**

This meta-analysis indicates that DM is positively correlated to EC. However, the results should be interpreted with caution because of the limitations on potential clinical confounding factors in each study included in this meta-analysis.

## 1 Introduction

It has been reported that esophageal cancer (EC) has been the 8th common cancer around the world.^[1]^ For example, in 2008 there were 480,000 newly diagnosed EC cases and 400,000 deaths were caused by EC, most of which occurred in China.^[2]^

EC has been a serious disease around the world. It is said that the rate of 5-year mortality will exceed 80% in patients with EC.^[3]^ Generally, EC has 2 types: esophageal squamous cell carcinoma (ESCC) and esophageal adenocarcinoma (EAC). In contrast with ESCC, EAC was the more common type.^[4]^ In the 1990s, more than 95% of esophageal malignancies were ESCC. However, the incidence of EAC increased approximately 6-fold in the United States from 1975 to 2001^[5]^ and a decreased incidence of ESCC has been seen in western countries.^[6]^ In addition, the survival rate of EC remains low, despite the rapid progress of diagnosis and treatment. Therefore, EC remains a seriously fatal disease around the world.

Prevention of EC seems to be very urgent. And identification of potential risk factors^[7]^ that affect the progression of EC would be important for prevention or early detection of EC. EC has 2 main types, and there are several significant differences in pathogenesis, tumor biology, and individual characteristics between ESCC and EAC. For ESCC, tobacco, dietary carcinogen exposure, and alcohol consumption are the known risk factors. Besides, Stroup et al^[8]^ also identified the infection of *Helicobacter pylori* as another potential risk factor. For EAC, gastro-esophageal reflux disease (GERD), white race, male gender, obesity, and smoking are regarded as established risk factors for EAC development.^[9]^ Nevertheless, many patients have not been exposed to these risk factors.

In recent years, the prevalence of diabetes mellitus (DM) was significantly increased around the world.^[10,11]^ Many studies have demonstrated that DM might be a risk factor for some types of cancer, such as hepatocellular carcinoma, breast cancer, endometrial cancer, bladder cancer, and kidney cancer.^[12–16]^ The detailed mechanisms may be explained by the effects of insulin and insulin-like growth factors (IGFs) axis on cells growth. A number of studies reported that IGFs axis could trigger intracellular signaling transduction involved in the development of cancers.^[17,18]^

In 1991, the association between type 2 diabetes mellitus (T2DM) and EC was first reported. After that, a number of studies regarding DM and EC susceptibility were conducted.^[1,16,19–29]^ However, results of different studies are inconsistent. This study aims to resolve the inconsistencies by incorporating some of the relevant studies investigating the exact number of EC patients in DM and non-DM groups.

## 2 Materials and methods

### 2.1 Search strategy

This meta-analysis was conducted according to Preferred Reporting Items for Systematic Reviews and Meta-analyses guidelines (PRISMA).^[30]^ All articles involving DM and EC were obtained from the English literature. A computerized literature search was implemented in PubMed, MEDLINE, and Web of Science databases till August 2016 with the following text word or Medical Subject Heading (MeSH) terms: (“diabetes mellitus” or “diabetes” or “DM” or “T2DM”) AND (“esophageal neoplasm” or “esophagus neoplasm” or “esophagus cancer” or “esophageal cancer” or “esophagus adenocarcinoma” or “esophageal adenocarcinoma” or “EC” or “EAC” or “ESCC”).

### 2.2 Study selection

The studies that met all the following criteria were brought into the meta-analysis: they were case–control studies; studies must have incidence of EC, EAC, or ESCC (or data to calculate them); there were odds ratio and 95% confidence interval (CI) in case–control studies; and patients diagnosed with type 1 diabetes mellitus (T1DM) were excluded from this analysis.

### 2.3 Ethics statement

As our study was a secondary analysis regarding human subject data published in the public domain, ethical approval was not necessary in this review.

### 2.4 Data extraction and quality assessment

Three investigators (BX, XHL, and XFZ) independently collected the following data from the included studies according to a predefined protocol: the first author's last name, publication time, country of the study, source, ethnicity (North America, Europe, and Asian), sample size (cases and controls), confounders or adjusted factors, the odds ratio and 95% CI, and solved disagreement by discussion.

Three investigators (BX, XHL, and CYL) independently accomplished the quality assessment according to the Newcastle–Ottawa Quality Assessment Scale (NOS).^[31]^ The NOS system consisted of 3 aspects: subject selection (0–4 points), comparability (0–2 points), and clinical outcome (0–3 points). And NOS scores ≥6 are identified as high-quality studies.

### 2.5 GRADE quality assessment

The quality of evidence based on the results of meta-analysis would be assessed by GRADE system (version 3.6). The GRADE system included 4 levels of evidence: high quality: further research may not change the effect of the credibility of the evaluation result; the medium quality: further research is likely to affect the curative effect of the credibility of the evaluation result and can change the results of the assessment; low quality: further studies are likely to influence the reliability of the efficacy evaluation, and this assessment is likely to change; and extremely low quality: any efficacy evaluation results are very uncertain. Two reviewers (BX and CZY) assessed quality independently and solved disagreement by discussion.

### 2.6 Statistical analysis

The incidence of EC in each study was regarded as a binary variable. Study-specific risk ratio (RR) was assessed by a fixed- or random-effect model^[32]^ using Cochrane Library software Review Manager (version 5.3 software). *I*^2^ value was adopted for the quantification of statistical inconsistency between studies due to heterogeneity, in which *I*^2^ < 30% demonstrated mild heterogeneity, *I*^2^ is between 30% and 70% showed moderate heterogeneity, and *I*^2^ > 70% suggested severe heterogeneity.^[33]^ Funnel plot analysis was used to assess publication bias using Stata software (version 12.0). *P* < .05 was considered statistically significant.

## 3 Results

**Figure 1:**
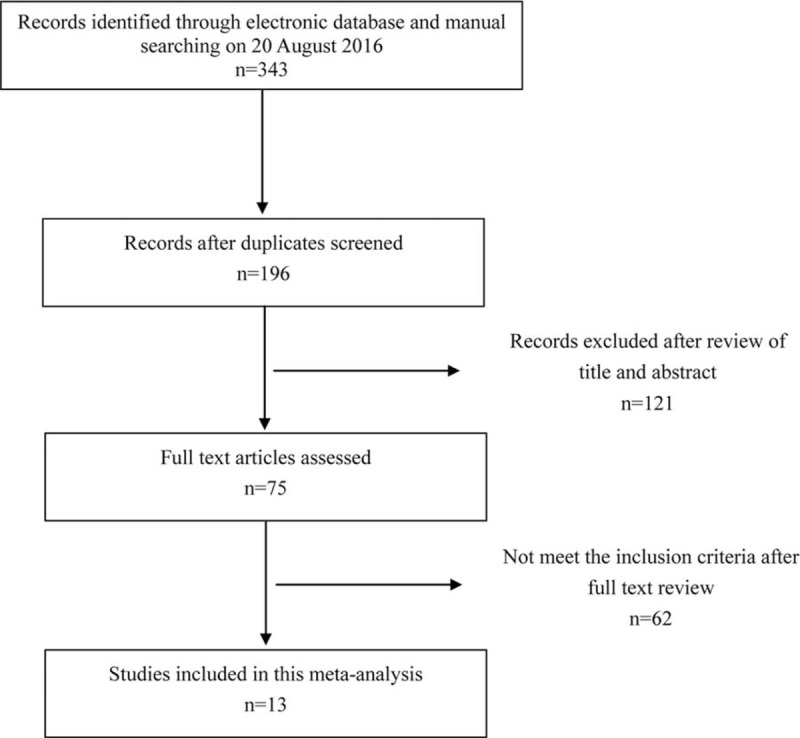
The publication selection process.

A total of 339 articles were identified during the initial search. After a careful review, studies on other cancers except EC in patients with DM were excluded, and 75 relevant articles were obtained for further evaluation. Finally, 13 studies^[1,16,19–29]^ were included in this meta-analysis (Fig. 1), including 20,611 cases and 177,186 controls. The detailed information of subjects in this study was listed in Table 1. In addition, the results of GRADE were shown in Table 2.

**Table 1:**
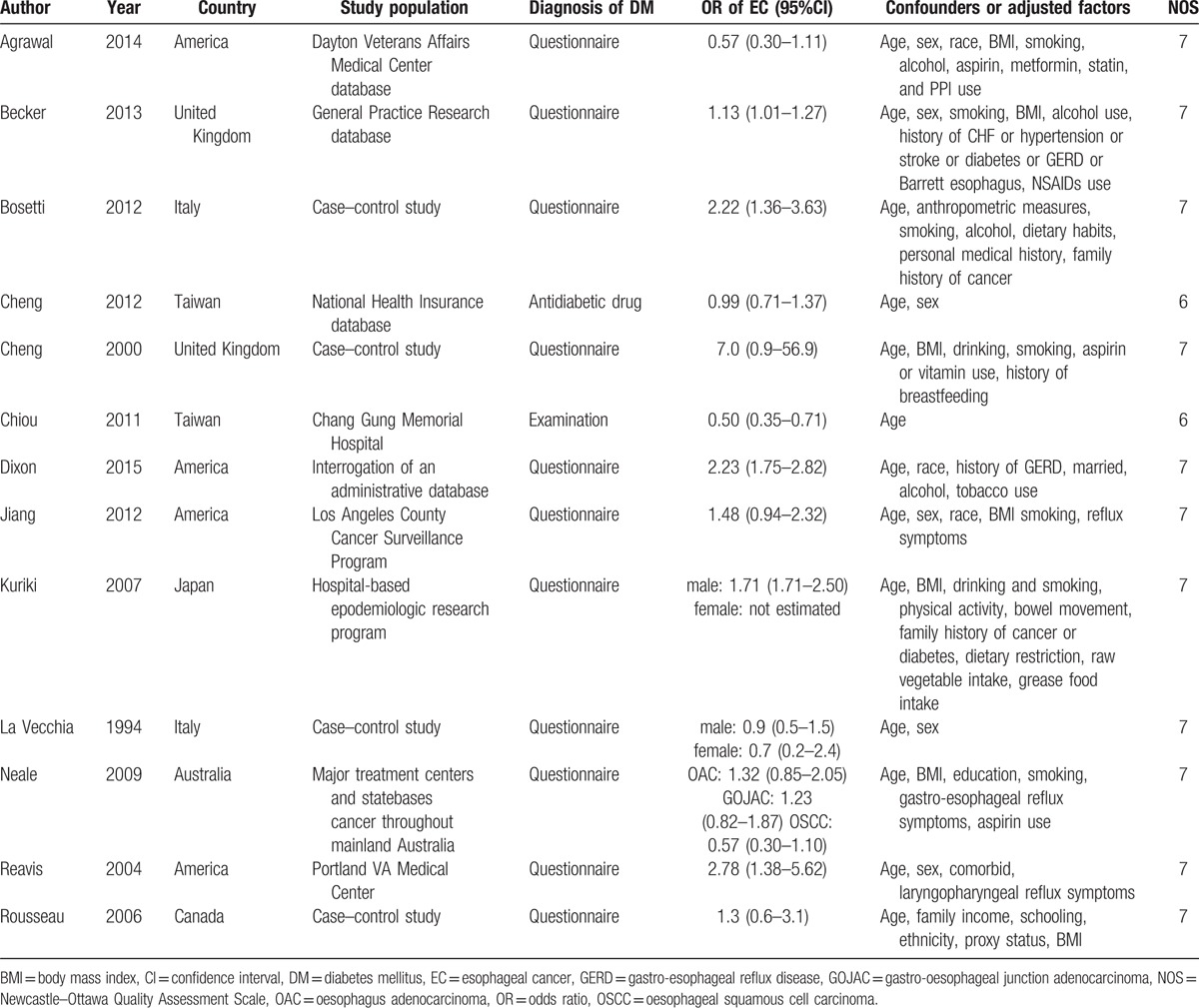
Summary of included studies.

**Table 2:**
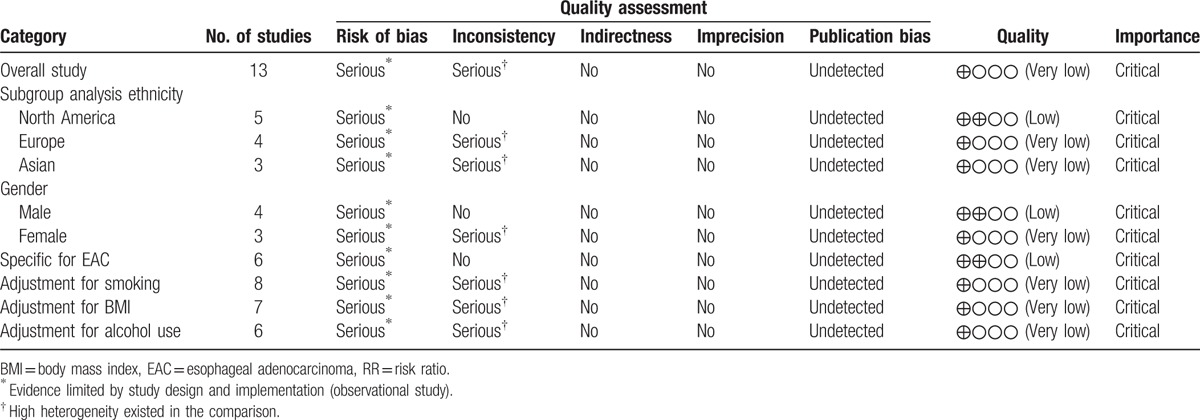
GRADE profile evidence of the included studies.

**Figure 2:**
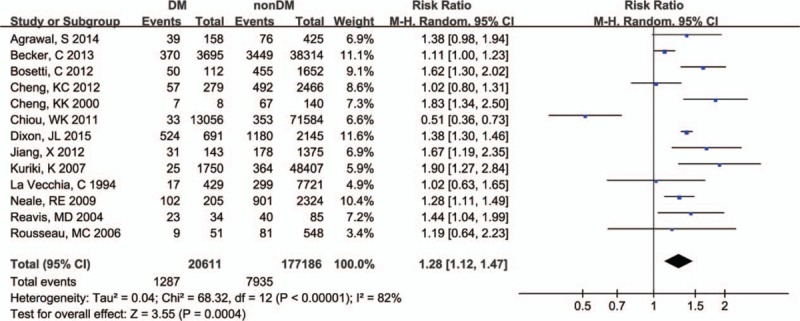
All included publications forest plot model.

Due to the significant heterogeneity (*I*^2^ = 82%; *P* < .001), we chose the random-effect model to estimate the pooled RR. The results showed a meaningful association between DM and EC, suggesting DM was a risk factor for EC (RR = 1.28, 95% CI: 1.12–1.47, *P* < .001) (Fig. 2). Subsequently, the subgroup analysis based on gender revealed that male was a significant risk factor for EC (RR = 1.53, 95% CI: 1.44–1.62, *P* < .001), but female was not (RR = 1.23, 95% CI: 0.41–3.69, *P* = .71) (Table 3). In addition, subgroup analysis based on ethnicity showed that DM was significantly correlated to EC in North American subjects (RR = 1.39, 95% CI: 1.31–1.47, *P* < .001) and in European subjects (RR = 1.37, 95% CI: 1.02–1.83, *P* = .04); however, no correlation was found in Asian subjects (RR = 0.98, 95% CI: 0.50–1.95, *P* = .96) (Table 3).

**Table 3:**
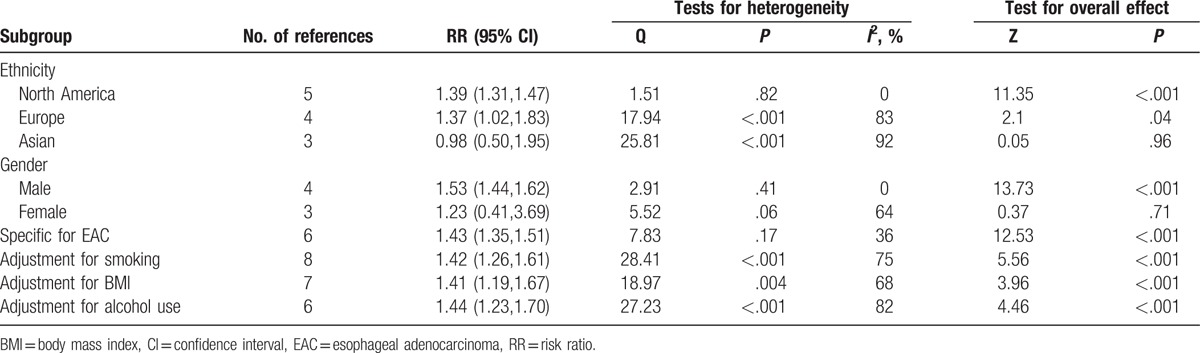
Subgroup analysis of relative risks for the association between diabetes mellitus and esophageal cancer risk.

When we limited the meta-analysis to the 6 studies specified for the subtypes of EAC,^[1,24,25,27,34,35]^ we found a positive association between EAC and diabetes (RR = 1.43, 95% CI: 1.35–1.51, *P* < .001) (Table 3). Obesity, smoking habits, and alcohol use are 3 of the most important confounders for the association between diabetes and EC risk. And we found a significant association between diabetes and EC (RR = 1.41, 95% CI: 1.19–1.67, *P* < .001) when controlled for body mass index with a meta-analysis including 7 studies. Similarly, a significant correlation was also found between diabetes and EC risk, when we limited the meta-analysis to the studies that controlled for smoking habits (RR = 1.42, 95% CI: 1.26–1.61, *P* < .001) or alcohol use (RR = 1.44, 95% CI: 1.23–1.70, *P* < .001).

**Figure 3:**
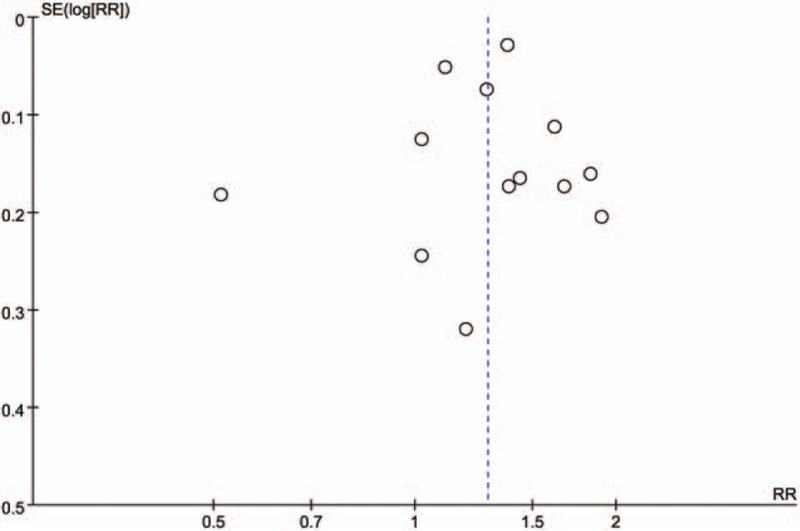
Funnel plot analysis of all included articles. Begg correlation test (*P* = .951) and Egger test (*P* = .742).

**Figure 4:**
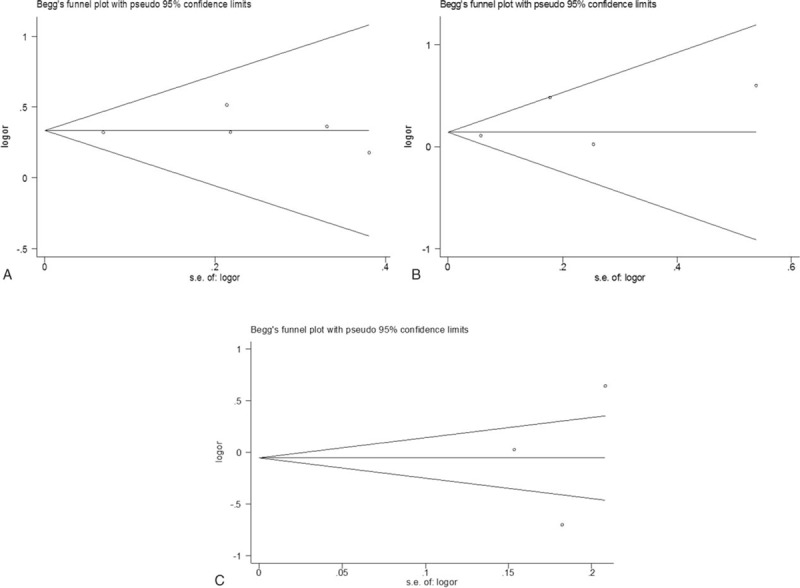
Funnel plot analysis of (A). Begg correlation test (*P* = 1.000) and Egger test (*P* = .740). Funnel plot analysis of (B). Begg correlation test (*P* = .734) and Egger test (*P* = .406). Funnel plot analysis of (C). Begg correlation test (*P* = 1.000) and Egger test (*P* = .804).

**Figure 5:**
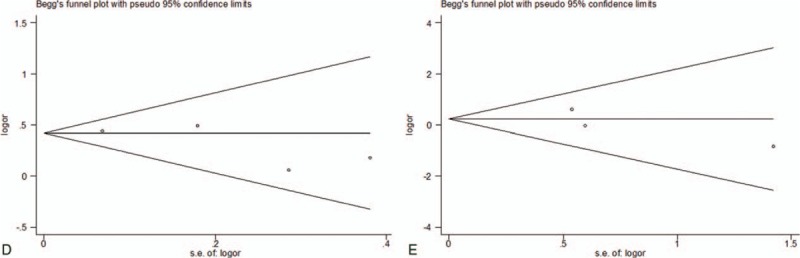
Funnel plot analysis of (D). Begg correlation test (*P* = .308) and Egger test (*P* = .286). Funnel plot analysis of (E). Begg correlation test (*P* = .296) and Egger test (*P* = .429).

**Figure 6:**
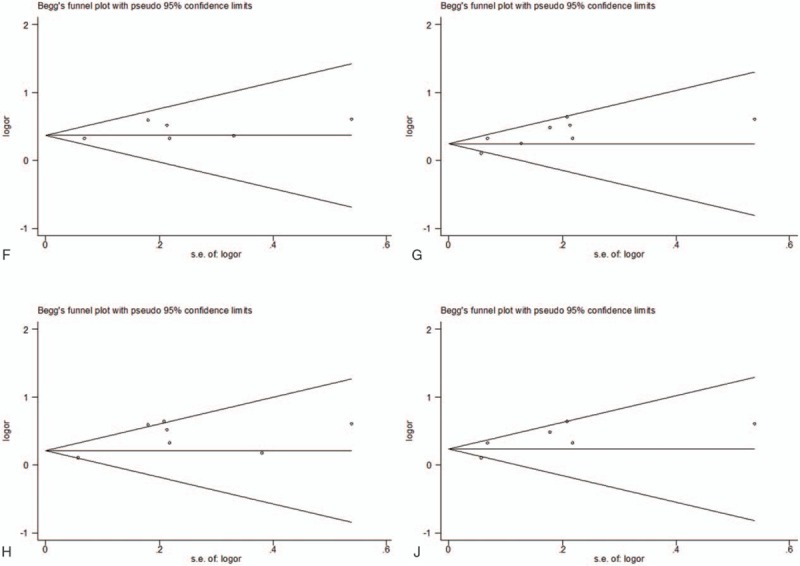
Funnel plot analysis of (F) Begg correlation test (*P* = 1.000) and Egger test (*P* = .191). Funnel plot analysis of (G). Begg correlation test (*P* = .711) and Egger test (*P* = .050). Funnel plot analysis of (H). Begg correlation test (*P* = .548) and Egger test (*P* = .050). Funnel plot analysis of (J). Begg correlation test (*P* = 1.000) and Egger test (*P* = .134).

Moreover, the publication bias analyses showed that the funnel plots seemed basically symmetric, and the results of the Begg test (*P* = .951) and the Egger test (*P* = .742) indicated that there was no publication bias in the present study (Fig. 3). Subsequently, the funnel plots based on ethnicity showed that there was no publication bias (Fig. 4A–C). And the funnel plots based on gender revealed that there was no publication bias (Fig. 5D, E). In addition, the funnel plots of specific for EAC (Fig. 6F), adjustment for smoking (Fig. 6G), adjustment for smoking (Fig. 6H), and adjustment for smoking (Fig. 6J) indicated that there was no publication bias.

## 4 Discussion

This meta-analysis suggested that diabetic group may have an approximately 28% increased risk of developing EC, which was consistent with a previous meta-analysis.^[14]^ The subgroup analysis indicated that in diabetic patients, gender and ethnicity affected the EC susceptibility, and male, North America subjects and Europe subjects were the important risk factors for EC.

As we all know, DM is a very common disease, and its prevalence has been significantly increased around the world.^[36]^ T2DM has a positive correlation with some types of human cancer, such as hepatocellular carcinoma, breast cancer, endometrial cancer, bladder cancer, and kidney cancer.^[12–16]^ However, the mechanism of how EC developed in patients with DM remains unclear, and there are several possible mechanisms below:

(1) Hyperglycemia is the connecting link between DM and cancer.^[37]^ Hyperglycemia can cause interaction of the crosstalk between oxidative stress and advanced glycation products (AGEs)/advanced glycation products receptor (RAGE) system. The crosstalk can activate cell signaling pathways that stimulate cellular growth and inhibit apoptosis,^[38]^ which could lead to cancer and cell invasion.^[39]^
(2) Type 2 diabetes has a correlation with hyperinsulinemia and insulin resistance. Hyperinsulinemia is associated with increased bioactive serum IGF-1 and can upregulate the concentration of IGF-1. And activation of the IGF-1 and insulin receptors results in intracellular signaling cascades in both the extracellular signal-related kinase (ERK) and phosphatidylinositol-3 kinase (PI3K) pathways, producing downstream mitogenic, antiapoptotic, and proangiogenic effects that may favor tumor growth in EAC.^[40]^ What is more, the study demonstrated that serum total and free IGF-1 levels are significantly increased among viscerally obese patients compared with normal weight controls, and among patients with EAC compared with Barrett esophagus (BE) and healthy controls.^[41]^
(3) Delayed gastric emptying commonly seen in DM may be another mechanism.^[42]^
(4) Gastric hypomotility is a key factor for reflux symptom development and progression which may lead to BE and EAC.^[43]^
(5) Obesity which is common in T2DM is an established risk factor for EAC, although the precise mechanism remains unknown. An emerging hypothesis is that metabolic aberrations accompanied with obesity lead to changes in hormones and cytokines, including insulin, and serum insulin may play a role in BE progression through a number of mechanisms.^[4,14]^ In a word, each of these mechanisms proves a metabolic abnormality in DM patients and could underlie the association between EC and DM.

In the present meta-analysis, we found that male with DM had an increased risk of EC. But the detailed mechanisms involved in this phenomenon are still unclear. As we all know, male had a predominant position among patients with GERD, BE, and EC, especially EAC.^[14]^ Although there is no study investigating this pathway within a single population so far, a meta-analysis by Cook et al^[44]^ has proved that BE, a potentially precancerous condition, has a pooled male/female sex ratio of 1.96/1 (95% CI: 1.77, 2.17/1); and EAC has a higher sex ratio (7–10:1) in most western countries.^[6]^ This difference of sex ratio might be in part explained by the gonadal hormones (for instance, estrogen and androgen), because previous studies demonstrated that compared to normal cells, the expression of sex hormone receptors has been upregulated in human EAC cells.^[45,46]^ In a word, more researches on the effects of gender on EC with DM needed to be done for underlying mechanisms and therapeutic interventions. In addition, we also found the North American and European subjects had an increased risk of EC compared with Asian subjects. This may partly result from genetic diversity among ethnicities. Furthermore, lifestyle (such as diet, smoking, drinking, etc) could inevitably play an important role in this process.^[14]^ What is more, the prevalence of obesity in western countries is higher than that in eastern countries, which is a risk factor of GERD that could lead to EAC.^[4,14]^

There are still several limitations in present meta-analysis. First, case–control studies are susceptible to recall and interviewer bias. And the assessment of exposure factors in the studies was based on “self-statement,” which would introduce bias into these studies and affect the real results. Second, the articles in the present meta-analysis did not distinguish T1DM from T2DM. Since the misclassification of diabetes exists, it will weaken the degree of the correlation between diabetes and EC risk. However, it is likely that the majority of DM patients in this meta-analysis are T2DM, as this disease is by far the most common form particularly in older persons and the incidence of T1DM is less than T2DM. Third, confounding factor is likely to be present. Unhealthy lifestyles (such as smoking and alcohol abuse) and obesity have been considered to increase the risk of cancers. Although most studies in this meta-analysis have controlled these lifestyle factors, not all interference factors could be completely ruled out. Moreover, no researches could exclude the effects of hypoglycemic drugs, which may increase or decrease cancer risks.^[47]^ Each of these may enhance difficulty to assess EC risk in DM patients, so deep-going researches based on these confounding factors are needed.

In this meta-analysis, there were 6 studies presenting results specific for EAC,^[1,24,25,27,34,35]^ which showed a significant positive correlation between diabetes and EAC risk. However, the results need to be interpreted with caution because of several reasons: The incidence of diabetes among controls was different from prevailing population estimates for each study; Five studies adjusted for GERD, which are the most important risk for EAC. And several studies did not control for smoking habits and body mass index, which are also risk factors for EAC. The association may reflect confounding by the 2 risk factors. Therefore, further researches are to be conducted on the correlation between DM and EAC.

According to the methodology of GRADE quality evaluation, the quality of the evidence was very low in overall study result and most subgroup analysis results, and there were several possible reasons: the weak study design was unlikely to explain all of the apparent benefit or harm, even though observational studies were likely to provide an overestimate of the true effect; the assessment of exposure factors in the studies was based on “self-statement,” which would introduce bias into these studies and affect the real results.

In conclusion, this meta-analysis indicates that DM may have an association with an increased EC risk. However, it is a pity that most of DM patients only focus on DM treatment other than prevention of cancers or screening for several types of cancers.^[48]^ Therefore, comprehensive therapeutic interventions should be seen in DM patients. As the association between DM and EC remains in dispute, a well-designed prospective study on the correlation between DM and EC is still to be warranted.
